# Therapeutic Advances of Curcumin and Nanocurcumin in Glioblastoma: Molecular Targets, Bioavailability, and Drug Delivery

**DOI:** 10.3390/nu18020194

**Published:** 2026-01-07

**Authors:** Md Ataur Rahman, Mahesh Kumar Yadab, Meser M. Ali

**Affiliations:** Department of Oncology, Karmanos Cancer Institute, Wayne State University, Detroit, MI 48201, USA

**Keywords:** curcumin, nanocurcumin, glioblastoma, blood–brain barrier, drug delivery, molecular targets

## Abstract

Glioblastoma (GBM), the most common, invasive, and chemoresistant form of adult primary brain cancer, is characterized by rapid cell proliferation, local invasiveness, and resistance to chemotherapy (e.g., temozolomide (TMZ)) and radiation therapy. Curcumin, a bioactive polyphenol derived from *Curcuma longa*, has exhibited exceptional anti-cancer properties, including anti-proliferative, pro-apoptotic, anti-inflammatory, and anti-angiogenic activities in a wide range of cancer models, including GBM. However, the clinical application of curcumin has been seriously limited by several challenges, including low water solubility, low bioavailability, rapid systemic clearance, and poor blood–brain barrier (BBB) penetration. To overcome these challenges, several nanocarrier systems to produce nanocurcumin have been developed, including liposomes, polymeric nanoparticles, solid lipid nanoparticles, dendrimers, and micelles. These nanoformulations improve the solubility, stability, systemic circulation, and target-directed delivery of curcumin to glioma cells, thereby resulting in a high level of accumulation in the glioma microenvironment. On the other hand, this work is devoted to the potential of curcumin and nanocurcumin for the treatment of GBM. The article provides a detailed review of the major molecular targets of curcumin, such as NF-κB, STAT3, PI3K/AKT/mTOR, and p53 signaling pathways, as well as recent advancements in nanotechnology-based delivery platforms that improve drug delivery across the BBB and their possible clinical translation. We also include a thorough examination of the issues, limitations, and potential opportunities associated with the clinical advancement of curcumin-based therapeutics for GBM.

## 1. Introduction

About 15% of all intracranial neoplasms are glioblastomas (GBM), sometimes referred to as glioblastoma multiforme, the most common and deadly type of primary malignant brain tumor in adults [1]. With a median survival of only 12 to 15 months and a 5-year survival rate below 10%, the prognosis for GBM patients is still poor despite advancements in surgical resection, radiation therapy, and chemotherapy-most notably with the DNA-alkylating drug temozolomide (TMZ) [2,3]. The highly aggressive nature of the tumor, its intrinsic and acquired resistance to treatment, its extensive infiltration into healthy brain tissue, and the protective function of the blood–brain barrier (BBB) [4], which limits the efficient delivery of most chemotherapeutic drugs, are all factors contributing to this dismal result. These treatment limits highlight the pressing need for new, safe, and efficient molecules that can overcome obstacles in drug delivery and target glioblastoma at the molecular level.

Because of its pleiotropic pharmacological properties, curcumin, a naturally occurring polyphenolic substance obtained from the rhizome of *Curcuma longa* (turmeric), has drawn a lot of interest in oncology research [5]. Curcumin has been shown in preclinical research to have anti-inflammatory, antioxidant, pro-apoptotic, anti-angiogenic, and anti-proliferative properties against glioblastoma and other malignancies [6]. Curcumin mechanistically alters several important signaling pathways, including PI3K/AKT/mTOR, NF-κB, STAT3, Wnt/β-catenin, Notch, and p53, that are linked to the pathophysiology of GBM [7]. Curcumin has the potential to be a multi-targeted drug in GBM treatment since it targets several nodes of tumor survival and resistance [8]. Curcumin has also been demonstrated to make glioma cells more sensitive to TMZ and radiation, which may have been used in combination therapy approaches [9,10].

However, curcumin’s physicochemical constraints have posed a substantial obstacle to its clinical translation. Because curcumin is hydrophobic, rapidly metabolized in the liver and intestinal wall, and has limited absorption, oral administration of this compound results in poor systemic bioavailability [11]. More significantly, its incapacity to efficiently traverse the blood–brain barrier limits its build-up in brain tumors, thus diminishing its therapeutic potential in neuro-oncology [12]. Recent studies have concentrated on creating delivery systems based on nanotechnology that encapsulate curcumin into nanocarriers, enhancing their solubility, stability, biodistribution, and permeability across the blood–brain barrier to overcome these constraints [13]. A range of nanocarriers, including polymeric nanoparticles, liposomes, dendrimers, micelles, and solid lipid nanoparticles, are used by nanocurcumin, the nano-formulated form of curcumin, to improve its pharmacokinetic and pharmacodynamic characteristics [14]. To enable active targeting of GBM cells via receptor-mediated endocytosis, these nanocarriers can be functionalized with targeting ligands (such as transferrin, folic acid, or RGD peptides) [15]. Furthermore, preferential accumulation at the tumor site can be made possible by engineering nanoparticles to take advantage of the increased permeability and retention (EPR) effect in the tumor vasculature. In vivo models of glioblastoma, several nanoformulations have shown enhanced tumor selectivity and BBB penetration [16]; several have also shown greater cytotoxicity against populations resistant to therapy and glioma stem-like cells.

The goal of this study is to present a thorough summary of the most recent developments in curcumin and nanocurcumin research for the treatment of glioblastoma. We start by providing an overview of the molecular pathways that curcumin uses to achieve its anticancer effects, with a particular emphasis on important signaling pathways linked to GBM. Next, we look at new developments in delivery systems based on nanotechnology that are intended to improve curcumin’s bioavailability and BBB permeability. Furthermore, we explore preclinical research that evaluates the possible synergy between curcumin and nanocurcumin when combined with conventional GBM treatments. Lastly, we discuss the present difficulties, constraints, and prospects for converting these discoveries into clinical uses. Curcumin and its nanoformulations may provide a revolutionary approach to the treatment of glioblastoma, a disease that continues to elude standard therapeutic approaches, by fusing molecular pharmacology with nanotechnology. New approaches to individualized, multi-modal cancer treatment are made possible by the combination of targeted medication delivery and natural product research; curcumin and nanocurcumin are positioned as top contenders in this new paradigm.

## 2. Curcumin’s Molecular Mechanisms and Targets in Glioblastoma

**Figure 1:**
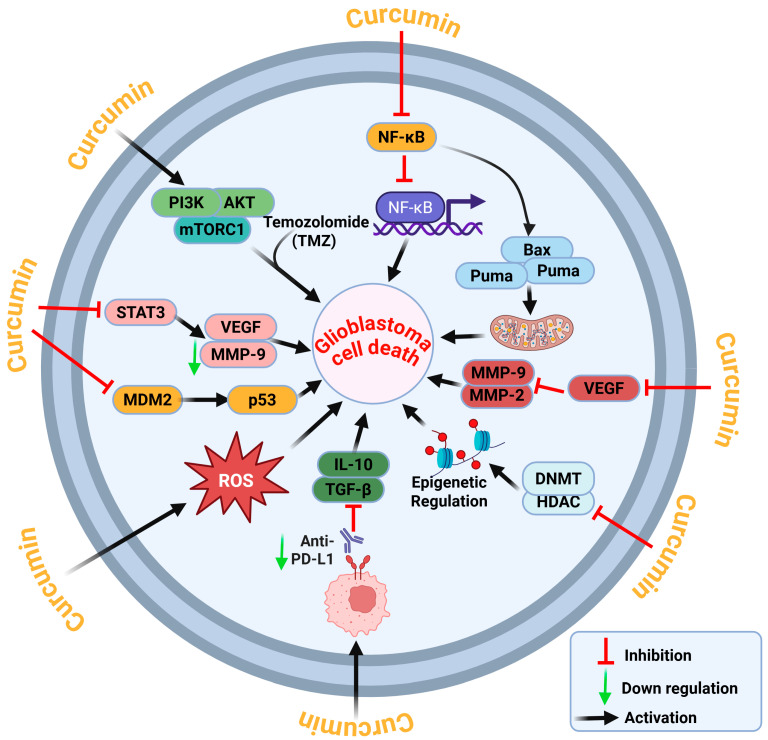
**Molecular mechanism of curcumin-mediated GBM cell death.** Curcumin hinders PI3K/AKT/mTOR and STAT3 signaling, diminishes NF-κB activation, and inhibits MDM2 to stabilize p53. Curcumin stimulates intrinsic apoptotic pathways through the activation of Bax and Puma, increases reactive oxygen species (ROS) production, and diminishes the expression of angiogenic and invasive markers, including VEGF and MMP-2/9. It modifies immunosuppressive cytokines (IL-10, TGF-β), diminishes PD-L1 expression, and regulates epigenetic modifiers (DNMT, HDAC) to reinstate tumor suppressor gene expression. These synchronized responses lead to glioblastoma cell apoptosis and increased susceptibility to temozolomide (TMZ) and Immunotherapeutics. The figure was created in BioRender.com by Md Ataur Rahman (2025).

By altering several signaling pathways that control cell division, apoptosis, angiogenesis, inflammation, and treatment resistance, curcumin has strong anticancer effects in glioblastoma. Curcumin’s pleiotropic action provides a clear benefit in the treatment of complex, heterogeneous malignancies like glioblastoma [17], in contrast to numerous monotherapeutic medicines that target a single component. Curcumin has anti-glioblastoma actions by multi-targeted regulation of essential oncogenic and tumor suppressor pathways [18]. Curcumin impairs the PI3K/AKT/mTOR and NF-κB pathways, thus diminishing tumor cell viability, inflammation, and proliferation. It activates p53 by downregulating MDM2, resulting in mitochondrial-mediated apoptosis through Bax and Puma. Curcumin further downregulates STAT3, VEGF, and MMPs, hence inhibiting angiogenesis and invasion. Moreover, it modulates reactive oxygen species (ROS), inhibits epigenetic enzymes (DNMT, HDAC), and reinstates the anti-tumor immune response by downregulating IL-10, TGF-β, and PD-L1 expression. These actions collectively induce glioblastoma cell death (Figure 1).

### 2.1. PI3K/AKT/mTOR Pathway Inhibition

Glioblastoma often exhibits hyperactivation of the PI3K/AKT/mTOR signaling pathway, which is essential for fostering cell survival, proliferation, and treatment resistance [19]. It has been demonstrated that curcumin inhibits the activation of this pathway by downregulating mTOR and AKT phosphorylation. Reduced protein synthesis, autophagy activation, and apoptotic cell death are the results of inhibiting this axis [20]. Furthermore, curcumin targets the PI3K/AKT/mTOR pathway to make glioblastoma cells more sensitive to temozolomide (TMZ), which may indicate a role for curcumin in combination therapy [21].

Curcumin exerts its actions mechanistically at multiple levels, including the inhibition of upstream PI3K activity, as well as the targeting of phosphatases such as PTEN, to restore negative regulation that is often lost in different GBM subtypes [22]. However, the preponderance of evidence is in vitro or in vivo xenograft studies, and the translational potential requires further validation in appropriately powered and designed clinical trials. Early phase nanocurcumin formulations show improved accumulation in the glioma microenvironment, as well as sustained inhibition of AKT/mTOR signaling in preclinical orthotopic models [6]; however clinical validation of these pharmacologic effects has yet to be demonstrated. Additionally, feedback activation and crosstalk with other pathways such as MAPK or STAT3 can counteract the therapeutic efficacy of curcumin, necessitating combinatorial strategies [23]. Future studies should focus on assessing pathway blockades in patient-derived GBM stem cells and the incorporation of predictive response biomarkers. This would elevate curcumin from a generally cytotoxic agent to a pathway specific, precision medicine in GBM treatment.

### 2.2. NF-κB Modulation

One important transcription factor that controls genes related to inflammation, tumor growth, and apoptosis resistance is signaling NF-κB. By inhibiting IκB kinase (IKK) activation, curcumin prevents NF-κB from translocating to the nucleus and, therefore, the expression of anti-apoptotic proteins like Bcl-2, Bcl-xL, and surviving [24]. This inhibition increases the cytotoxicity of common chemotherapeutic drugs and restores apoptotic signaling. Curcumin, on a mechanistic level, can repress upstream inducers of NF-κB, such as TNF-α and IL-1β and prevent ROS-mediated NF-κB activation. Preclinical studies in orthotopic GBM and GSCs have confirmed this and show that curcumin is able to reduce NF-κB DNA-binding and NF-κB-regulated downstream cytokines, including IL-6 and COX-2 [25].

However, much of this preclinical data has been left largely to speculation in the clinic due to low levels of curcumin brain bioavailability. While there have been successful uses of nanocurcumin for improved NF-κB inhibition in glioma preclinical models, there has been no correlating pharmacokinetic-pharmacodynamic data or dose-optimization in humans. Additionally, given the NF-κB’s role in both immune regulation and cell survival, further understanding and careful targeting will be important, specifically within the context of combination immunotherapy [26]. Therefore, it will be important in future studies to see if the inhibition of NF-κB by curcumin offers long term benefits in the varying microenvironments of GBM or if there are resistance mechanisms that compromise its efficacy.

### 2.3. STAT3 Activation Suppression

Glioblastoma is constitutively active for signal transducer and activator of transcription 3 (STAT3), which aids in angiogenesis, tumor development, and immune evasion [27]. It has been demonstrated that curcumin efficiently inhibits STAT3 phosphorylation, which lowers the production of downstream targets like VEGF, cyclin D1, and MMP-9 [28]. In addition to preventing GBM cell migration and proliferation, this inhibition hinders tumor angiogenesis. Mechanistically, curcumin is shown to inhibit upstream modulators including IL-6, JAK2, and EGFR which could indicate broad interference in the network [29]. In preclinical studies, in U87MG cells and primary GBM cells, STAT3 signaling is reduced after treatment with curcumin or nanocurcumin, often in combination with TMZ or radiation with synergistic effects [30].

However, these studies are limited, and challenges remain to transfer the inhibition of STAT3 into long-term therapeutic outcomes due to the intratumoral heterogeneity of STAT3 expression and activation and also due to the possible activation of other pathways as compensatory mechanism (for example, PI3K or NF-κB). In addition, while nanocarrier systems are found to have better STAT3 inhibition in in vitro and xenograft models, the information about their ability to cross the blood–brain barrier and to selectively target the glioma stem-like cells is only hypothetical [31]. A critical analysis of the delivery systems (liposomes vs. dendrimers) for STAT3 modulation would be beneficial to future studies in combination immunotherapy or anti-angiogenic strategies for the therapy of resistant subtypes of GBM.

### 2.4. p53-Mediated Apoptosis Activation

Curcumin has demonstrated the ability to repair and activate p53-mediated apoptotic signaling in glioblastoma cells. Curcumin enhances the stability and nuclear accumulation of wild type p53, resulting in the transcriptional activation of pro-apoptotic genes including Bax, Puma, and Noxa [32]. This then facilitates mitochondrial outer membrane permeabilization (MOMP), the release of cytochrome c, and the activation of caspase-9 and caspase-3, resulting in intrinsic apoptotic cell death [33]. In cells harboring mutant p53, curcumin may induce p53-independent apoptosis via other mechanisms, including the suppression of NF-κB and AKT signaling pathways [34]. Furthermore, curcumin can inhibit the production of MDM2, a negative regulator of p53, thus augmenting p53 stability. Through these pathways, curcumin reactivates the p53 pathway and induces apoptosis, considerably enhancing its anticancer efficacy in glioblastoma [35].

Of note, most of the data supporting this mechanism is derived from in vitro GBM studies and xenograft studies using p53-wild-type cell lines. In p53-mutant or p53-deficient GBM cells, curcumin appears to induce apoptosis through p53-independent mechanisms, including the inhibition of NF-κB and AKT signaling, mitochondrial stress, and accumulation of ROS [36]. While this data highlights the versatility of curcumin across GBM subtypes, the restoration of mutant p53 is largely speculative and requires further validation [37]. Additionally, the different p53 mutational landscapes and the development of adaptive resistance may limit long-term efficacy. Nanocurcumin formulations have shown improved intracellular delivery and sustained p53 activation in preclinical models [38], yet the clinical validation of these pharmacologic effects is lacking. Future studies should incorporate patient-derived GBM models and biomarker-based stratification to determine which p53 contexts are more amenable to curcumin treatment.

### 2.5. Reactive Oxygen Species (ROS) Regulation

Curcumin exerts a bifunctional modulation of ROS, functioning as an antioxidant in non-pathological cells while displaying pro-oxidant activities in malignant cells, including glioblastoma. In GBM cells, curcumin can trigger an overproduction of reactive oxygen species through mitochondrial dysfunction, endoplasmic reticulum stress, and NADPH oxidase activation [39]. This overproduction causes DNA damage, lipid peroxidation, and protein oxidation, which when exceeding cellular antioxidant capacity (e.g., glutathione, catalase), may push the cellular redox balance towards oxidative stress-mediated cell death. Importantly, ROS act as an upstream regulator of autophagy, MAPK cascades, and p53 stabilization, suggesting that curcumin-induced oxidative stress not only directly promotes apoptosis in GBM cells but may also potentiate other apoptotic pathways [40].

Evidence from preclinical studies, such as increased malondialdehyde (MDA) levels and reduced superoxide dismutase (SOD) and glutathione peroxidase (GPx) activity in GBM models after curcumin treatment [41], has been demonstrated. However, some data implies a variability contingent on dosage and context, highlighting the need for in vivo pharmacodynamic optimization. Furthermore, while ROS modulation is critical to curcumin’s mode of action, its relationship with autophagy and mitochondrial-mediated apoptosis must be further elucidated through systems biology approaches [42]. The redox-sensitive transcription factors (e.g., Nrf2, HIF-1α) that curcumin could modulate in the GBM microenvironment are poorly understood and should be prioritized in future translational research.

### 2.6. Anti-Invasive and Anti-Angiogenic Qualities

By suppressing vascular endothelial growth factor (VEGF) and blocking matrix metalloproteinases (MMP-2 and MMP-9), which are necessary for the breakdown of extracellular matrix and tumor invasion, curcumin inhibits angiogenesis [43]. The tumor’s blood supply is restricted by these anti-angiogenic effects, which lowers the tumor’s ability for growth and metastasis. In addition, curcumin suppresses synthesis and enzymatic activity of matrix metalloproteinases MMP-2 and MMP-9, which have been involved in extracellular matrix degradation and basement membrane penetration during tumor cell invasion [44]. This may be mediated by inhibition of NF-κB and AP-1, which are transcription factors that regulate MMPs.

Preclinical studies using orthotopic GBM xenograft models and Matrigel plug assays have shown that curcumin significantly reduces microvessel density and invasive proliferation [45]. Dose-dependency was found in many cases and effects were often enhanced when curcumin was administered by nanoparticle delivery. There are limitations to the translation of these findings to the clinical setting. While many in vitro and preclinical studies demonstrate significant anti-angiogenic activity, curcumin’s short half-life and poor biodistribution to the brain have prevented translation to the clinic. Moreover, the relative contributions of curcumin-induced autophagy vs. direct MMP inhibition to block invasion need to be determined in more mechanistically oriented studies using specific pathway inhibitors and CRISPR models [46].

### 2.7. Glioblastoma Stem Cells (GSCs) Activation

One important factor contributing to tumor recurrence and treatment resistance is glioblastoma stem cells (GSCs). According to recent research, curcumin can decrease important stemness markers including CD133, Nestin, and Sox2 to target GSC populations [47]. Additionally, curcumin inhibits GSCs’ ability to self-renew and encourages differentiation, which lowers the tumorigenic potential of these cells [48]. GSCs are thought to play a critical role in tumor recurrence, chemoresistance, and radioresistance in GBM due to their self-renewal and survival capabilities [49]. Targeting GSCs has become a promising strategy to augment therapeutic efficacy. Curcumin’s ability to extensively regulate oncogenic signaling pathways provides a rationale for its potential impact on GSC maintenance and survival [50]. Curcumin potently inhibits the PI3K/AKT/mTOR signaling pathway, which promotes GSC proliferation, survival, and metabolic plasticity [51]. By blocking this pathway, curcumin can reduce GSC viability and induce autophagy-dependent or apoptotic cell death [52]. Curcumin has also been shown to inhibit the activity of STAT3, a key transcription factor involved in GSC stemness, immune evasion, and tumorigenicity [28]. Additionally, curcumin exhibits significant inhibition of NF-κB signaling, which regulates GSC survival and inflammatory cytokine production, thereby increasing therapeutic sensitivity [6]. However, the poor solubility and bioavailability of natural curcumin limit its efficacy against GSCs. Nanocurcumin formulations, including liposomes, dendrimers, and polymeric nanoparticles, have been developed to enable better delivery across the blood–brain barrier and enhance targeted accumulation in the glioma microenvironment [53]. These nanoformulations enhance intracellular uptake and sustained release of curcumin, leading to a significant increase in its anti-GSC efficacy. Therefore, nanocurcumin emerges as a promising therapeutic option to tackle GSC-mediated resistance in GBM.

### 2.8. Autophagy Modulation

**Figure 2:**
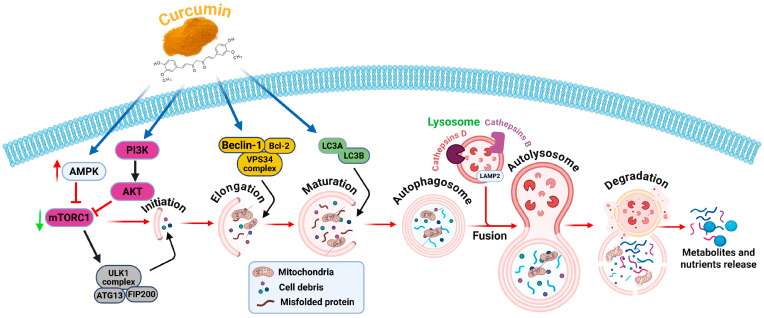
**Effect of curcumin on autophagy-mediated cell death in glioblastoma.** Curcumin regulates essential signaling pathways associated with autophagy control to promote autophagy-mediated apoptosis in GBM cells. It activates AMPK and inhibits the PI3K/AKT/mTOR signaling pathway, hence facilitating the beginning of autophagy mediated by the ULK1 complex. Curcumin augments the functionality of the Beclin-1/VPS34 complex and the LC3 conjugation system (LC3A/LC3B) during the elongation and maturation phases of autophagosomes. Autophagosomes subsequently combine with lysosomes, creating autolysosomes in which intracellular components are destroyed by cathepsins B and D. The process yields metabolites and nutrients, facilitating autophagic flow and cellular death. The figure was created and modified using the BioRender.com online commercial platform.

Autophagy has two functions in GBM: it can either cause autophagic cell death or increase cell survival under stress. By reorienting this equilibrium in favor of autophagy-dependent cell death (ADCD) [54], curcumin achieves its therapeutic effects. Curcumin functions by activating AMP-activated protein kinase (AMPK), which in turn suppresses mTOR, a crucial autophagy negative regulator [55]. By activating the ULK1 complex, this inhibition makes it easier for autophagy to begin. Additionally, curcumin inhibits the PI3K/AKT/mTOR signaling axis, which lowers the survival and multiplication of tumor cells [56]. Additionally, it encourages autophagosome buildup and lysosomal breakdown of misfolded proteins and damaged organelles, making GBM cells more vulnerable to stress-induced death [30]. Curcumin also affects the expression of Beclin-1, LC3B, and p62/SQSTM1, which are important indicators of autophagy flux and induction [57] (Figure 2). Crucially, by increasing autophagic flow in glioma stem-like cells, which are generally resistant to chemotherapy and radiation-curcumin can overcome therapeutic resistance [6]. Curcumin is therefore a prospective therapeutic approach to improve treatment efficacy and reduce drug resistance in glioblastoma due to its multi-targeted activity on autophagy-related signaling pathways.

### 2.9. Epigenetic Regulation

Curcumin functions as an inhibitor of histone deacetylase (HDAC) and DNA methyltransferase (DNMT) [58]. It reactivates tumor suppressor genes and silences oncogenes via modifying histone acetylation and DNA methylation patterns, which is especially significant in epigenetically dysregulated malignancies such as GBM [59]. Curcumin also could modulate epigenetic modifications, through regulation of chromatin remodeling and DNA methylation. As GBM is a tumor in which epigenetic silence plays a major role in its pathogenesis, this is relevant. Curcumin inhibits HDAC1 and HDAC3 in vitro, which leads to hyperacetylation of histone H3 and H4 and activation of tumor suppressor genes p21/WAF1/CIP1 and PTEN [60]. In parallel, curcumin represses DNMT1 and DNMT3b, resulting in hypomethylation of CpG islands and re-expression of the RASSF1A and MGMT genes that are hypermethylated in GBM [61]. In preclinical models of GBM (U87 and U251 cells), epigenetic reprogramming by curcumin was associated with reduced proliferation, increased temozolomide sensitivity, and restoration of apoptotic potential [62]. In an orthotopic model, intranasal treatment of curcumin nanoparticles increased histone acetylation and reduced tumor burden. However, it has not been directly proven whether epigenetic alterations are directly responsible for the above effects, or are merely secondary to blockade of upstream signaling (for example, PI3K/AKT or NF-κB). It is further been suggested that curcumin may also regulate non-coding RNAs (e.g., miR-21, miR-200c), which add another layer of epigenetic complexity [63]; however, this is still not well understood. Future studies should focus on ChIP-Seq and epigenome editing, to directly link curcumin treatment to specific epigenetic changes in GBM.

### 2.10. Immunomodulation

Curcumin alters the tumor immune microenvironment by reducing PD-L1 expression and suppressing immunosuppressive cytokines, including TGF-β and IL-10 [64]. It rejuvenates T-cell function and may enhance the efficacy of immune checkpoint inhibitors in glioblastoma immunotherapy [65]. Curcumin has demonstrated pronounced immunomodulatory activity in the GBM TME, which contributes to its potential to alleviate the immunosuppressive milieu that supports tumor growth and treatment resistance. Mechanistically, curcumin downregulates the expression of immunological checkpoint molecules like PD-L1 by targeting upstream signaling pathways such as STAT3 and NF-κB [66]. This leads to enhanced cytotoxic T lymphocyte (CTL) activity and restoration of T-cell-mediated immune surveillance. Additionally, curcumin suppresses the secretion of immunosuppressive cytokines like TGF-β and IL-10 by tumor-associated macrophages (TAMs) and myeloid-derived suppressor cells (MDSCs), reversing immunosuppressive polarization [67].

Preclinical studies in GL261 and U87 mouse models have reported that curcumin treatment increases CD8+ T-cell infiltration, reduces regulatory T cells (Tregs), and downregulates M2 macrophage markers, suggesting its potential to enhance the efficacy of immune checkpoint blockade therapies such as anti-PD-1/PD-L1 [66]. The translation of curcumin into GBM immunotherapy regimens is currently preclinical, with limited in vivo evidence on its effects on long-term survival or induction of immunological memory responses [68]. While nanocurcumin formulations are being explored to enhance brain delivery, only a few studies have explicitly evaluated the immunomodulatory effects in orthotopic GBM models. Further investigation is needed to validate curcumin’s immunological impact in patient-derived xenografts or immune-competent models, preferably in combination with established or emerging immunotherapies.

## 3. Curcumin Delivery Using Nanocarrier Systems for GBM Treatment

**Figure 3:**
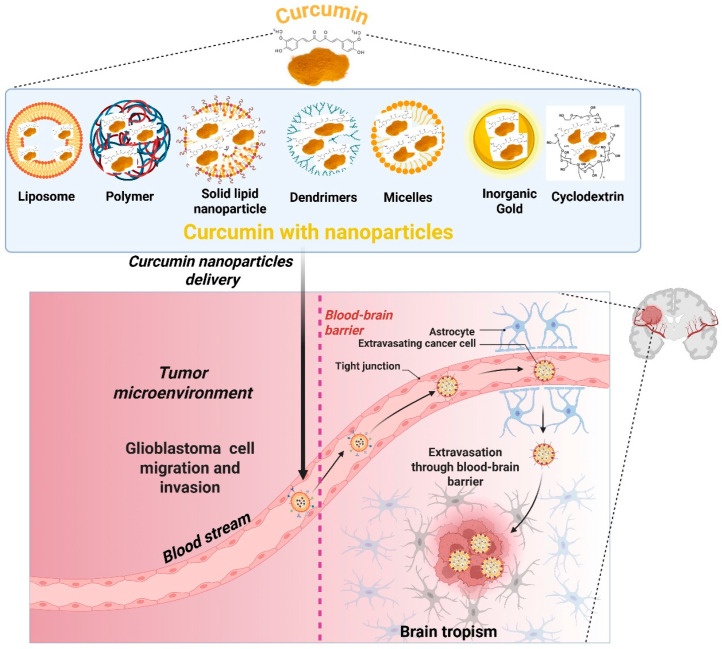
**Curcumin delivery using nanocarrier systems for glioblastoma treatment.** To overcome curcumin’s limited bioavailability and restricted blood–brain barrier permeability, numerous nanocarrier systems have been developed, including liposomes, polymers, solid lipid nanoparticles, dendrimers, micelles, gold-based inorganic nanoparticles, and cyclodextrins. These nanoformulations improve the solubility, stability, and circulation duration of curcumin. Upon systemic dosing, curcumin nanoparticles traverse the bloodstream, penetrate the blood–brain barrier through tight junction regulation or receptor-mediated transcytosis, and concentrate within the brain tumor microenvironment. This tailored administration promotes curcumin absorption by glioblastoma cells, increases therapeutic effectiveness, and minimizes off-target damage, hence facilitating the prevention of GBM cell invasion and proliferation. The figure was created and modified using the BioRender.com online commercial platform.

Curcumin’s limited permeability across the blood–brain barrier, fast metabolism, and poor aqueous solubility are some of the biggest obstacles to turning it into a successful treatment for glioblastoma [4]. The precise application of curcumin-encapsulated nanoparticles can overcome the BBB and efficiently target glioblastoma within the tumor microenvironment. Nanocarrier systems-comprising liposomes, polymers, solid lipid nanoparticles, dendrimers, micelles, inorganic gold particles, and cyclodextrins-designed to optimize curcumin delivery. The mechanism by which these nanoformulations enter systemic circulation, traverse the blood–brain barrier by receptor-mediated transport, and accumulate in the brain parenchyma. Upon traversing the blood–brain barrier, curcumin nanoparticles localize at tumor locations, impede glioblastoma cell migration and invasion, and demonstrate therapeutic efficacy, signifying a prospective strategy for targeted GBM treatment (Figure 3). Curcumin’s bioavailability and systemic circulation time are improved by nanocarriers, which also help with tumor-specific targeting and better distribution across the blood–brain barrier [69]. For the delivery of curcumin in GBM, a variety of nanocarrier systems have been created and refined; each has distinct physicochemical characteristics and therapeutic potential [6]. These consist of solid lipid nanoparticles, liposomes, dendrimers, micelles, polymeric nanoparticles, and inorganic nanoparticles. Below is a thorough explanation of each.

### 3.1. Liposomes

Liposomes are spherical vesicles with an aqueous core surrounded by one or more phospholipid bilayers. The lipophilic bilayer can be loaded with curcumin to increase its solubility and stability [70]. Formulations of liposomal curcumin improve biocompatibility and systemic administration [71]. Surface alterations using ligands like RGD peptides or transferrin can enhance tumor targeting and promote receptor-mediated transcytosis across the blood–brain barrier [72]. In preclinical models, studies have demonstrated that liposomal curcumin dramatically lowers tumor volume and glioma cell viability. Curcumin can be co-encapsulated with other drugs (siRNA, TMZ) in liposomes to target multiple pathways and for synergistic effects, thus increasing the mechanistic relevance [73]. Surface PEGylation can be used to prolong circulation half-life, and pH- or enzyme-sensitive liposomes may be used to provide triggered release in the acidic tumor microenvironment present in gliomas [74]. While preclinical data have shown encouraging results, most studies have used subcutaneous or ectopic models, and few have assessed pharmacokinetic profiles or survival benefits in orthotopic GBM models. In future studies, it will be important to distinguish between passive and active targeting, and to directly compare liposomes to newer delivery strategies to accurately gauge translational potential.

### 3.2. Nanoparticles of Polymers

To create polymeric nanoparticles for curcumin delivery, biodegradable polymers such chitosan, PEG-based compounds, and PLGA (poly(lactic-co-glycolic acid)) have been used extensively [75]. These nanoparticles can be functionalized with targeted ligands or antibodies, provide sustained drug release, and shield curcumin from enzymatic degradation [76]. For instance, PLGA-curcumin nanoparticles have demonstrated improved apoptotic activity through suppression of the PI3K/AKT pathway and increased absorption by glioma cells [77].

Studies have shown these polymeric nanoparticles to enhance intracellular retention and promote lysosomal escape, thus enhancing curcumin’s potential to modulate survival signaling and induce mitochondrial dysfunction [78]. Promising results have been observed in terms of tumor remission and prolonged survival in preclinical studies; however, these outcomes are often based on heterotopic xenografts or in vitro models that do not adequately replicate the complexity of the blood–brain barrier. There is a novel dual-loaded polymeric system being developed to co-load curcumin with either chemotherapeutics or siRNAs for a synergistic effect, however its readiness for clinical application requires validation in orthotopic GBM models [79]. Additionally, the kinetics of polymer degradation, potential immunogenicity, and reproducibility during scale-up are currently underexplored hurdles that need to be addressed before clinical translation.

### 3.3. A Solid Lipid Nanoparticle (SLN)

Solid lipids, which stay solid at body temperature, make up SLNs, which are submicron colloidal transporters [80]. They provide curcumin with superior stability, biocompatibility, and defense against oxidative deterioration. SLNs have been demonstrated to increase BBB permeability, and both in vitro and in vivo, curcumin-loaded SLNs dramatically reduce glioma cell proliferation and angiogenesis [81]. In GBM models, co-delivery of curcumin with other therapeutic drugs, such as TMZ, using SLNs has demonstrated encouraging synergistic benefits [82].

Mechanistic investigations reveal that SLN-loaded curcumin reduces pro-angiogenic factors, including VEGF and HIF-1α, and abrogates expression of EMT markers and MMP-9, thus, limiting tumor invasion and angiogenesis [83]. Some studies also report increased cellular uptake via clathrin-mediated endocytosis and improved escape from lysosomes, enabling successful intracellular delivery. However, these findings are mostly preclinical in nature, and the pharmacokinetics of SLNs in vivo in humans have not been established [84]. The toxicology of the surfactants and stabilizers used for solid lipid nanoparticle preparation also needs further investigation. Furthermore, reproducibility of SLN preparations, especially in cases of dual-drug loading or surface modification, is a translational challenge. Despite the potential of SLNs for GBM treatment, additional comparative studies and optimization for clinical-grade production are required to validate their therapeutic potential.

### 3.4. Dendrimers

Dendrimers are tree-like, extremely branching polymers with numerous surface functions and distinct topologies. Their distinct architecture enables accurate targeting and great drug loading efficiency. It is possible to design curcumin-conjugated dendrimers, especially those based on PAMAM (polyamidoamine), to penetrate the blood–brain barrier and deliver the medication precisely to tumor locations [85]. Additionally, dendrimers present the possibility of imaging-guided therapy and dual-drug delivery [86]. Mechanistically, dendrimer-curcumin conjugates have been shown to inhibit PI3K/AKT/mTOR and NF-κB signaling, induce autophagy, and trigger cell death in glioma models [87]. Some have been shown to induce mitochondrial dysfunction specifically in glioblastoma stem cells but not in normal astrocytes. Dual-drug dendrimer complexes (curcumin + TMZ/doxorubicin) have shown synergistic effects in preclinical GBM spheroid models [88]. However, many challenges remain. The cytotoxicity of some dendrimer generations, especially when lacking surface PEGylation, raises safety concerns [89]. While imaging-guided dendrimer systems have the potential to be theranostic, most remain at early stages with no long-term toxicity or pharmacokinetic data in humans. As such, while dendrimers hold great promise for translation, especially in the area of targeted multimodal therapy, their clinical translation will depend on careful structural optimization and consistency of safety profiles.

### 3.5. Micelles

Self-assembling amphiphilic nanostructures with a hydrophilic shell and a hydrophobic core are called polymeric micelles. They can improve the solubility of hydrophobic medications, like curcumin, in aqueous settings by encapsulating them. Micelles coated with curcumin have shown enhanced permeability and retention (EPR) effects that lead to higher tumor formation and improved cytotoxicity against glioma cells [90]. For glioblastoma-specific delivery, functionalized micelles that target EGFR or CD44 have been used [91]. These micelles have been shown to improve endocytosis-mediated intracellular delivery, leading to increased intracellular curcumin levels in cancer cells and more effective inhibition of oncogenic signaling pathways such as PI3K/AKT and NF-κB [92]. Functionalized micelles, particularly those targeting receptors like EGFR or CD44, allow for the selective targeting of glioblastoma and glioma stem-like cells, reducing off-target effects [91]. Preclinical data also suggests curcumin micelles may modulate autophagy and promote apoptosis in 3D GBM spheroids, a potential mechanism that needs further study [93]. Despite these promising developments, clinical translation is hampered by concerns over polymer biocompatibility, long-term circulation stability, and immunogenicity of the targeting ligands. Many studies remain in vitro or small animal stages, necessitating comprehensive comparative studies and scalable GMP-grade manufacturing for future clinical translation.

### 3.6. Nanoparticles That Are Inorganic

Inorganic nanoparticles, including gold, silica, and iron oxide-based magnetic nanoparticles, offer platforms for drug delivery, imaging, and theranostic capabilities in the treatment of glioblastoma [94]. The tunable size, shape, surface chemistry, and intrinsic physical properties of inorganic nanoparticles allow for enhanced conjugation of curcumin with increased stability, preferential distribution, and controlled release. Curcumin-conjugated gold nanoparticles (AuNPs) have shown enhanced cellular uptake and synergistic benefits, including photothermal ablation and radiosensitization [95]. These theranostic platforms can improve local control while reducing systemic toxicity. Magnetic nanoparticles, such as superparamagnetic iron oxide (SPION), can be used for image-guided delivery by magnetic resonance imaging (MRI) and can be further designed to undergo magnetically induced hyperthermia, which can augment curcumin cytotoxicity via apoptosis mediated by heat stress [96].

Mesoporous silica nanoparticles have an increased surface area and drug-loading potential and can be co-functionalized with BBB-targeting ligands for enhanced brain penetration [97]. These advancements have shown great potential for multimodal theranostic platforms that combine both diagnosis and therapy, as well as the ability to monitor these processes in real time. However, there are still challenges regarding long-term biosafety, clearance, and immune response. Most of the available data are derived from in vitro studies or small animal models and have not yet been validated in human settings. Further studies are required to evaluate the biodistribution, toxicity, and comparative efficacy of these delivery systems in more advanced preclinical GBM models to justify clinical relevance.

### 3.7. Techniques for Penetration of the Blood–Brain Barrier (BBB)

Through passive diffusion, adsorptive-mediated transcytosis, or receptor-mediated transcytosis, nanocarriers can be engineered to traverse the blood–brain barrier [98]. Nanoparticles can take over natural transport systems by functionalizing with ligands that target receptors like the insulin receptor, transferrin receptor (TfR), or LRP1 [99]. To aid BBB transit, surfactants such as polysorbate 80 and cell-penetrating peptides have also been used [100]. In animal models, these methods have demonstrated exceptional effectiveness in delivering curcumin to brain tumor locations. In addition, prolonging circulation time and BBB penetration with surfactants (e.g., polysorbate 80) or PEGylation mimics natural transport. Nanoparticles could be linked to cell-penetrating peptides (CPPs) such as TAT or penetratin to facilitate cellular uptake through tight junctions [101]. In recent preclinical studies, coating solid lipid nanoparticles with polysorbate-80 or angiopep-2-modified PLGA nanoparticles resulted in significantly increased curcumin levels in glioma with reduced off-target accumulation [102].

However, most of these strategies are still at the preclinical stage, and clinical translation faces challenges such as immune recognition, batch-to-batch consistency, and safety of repeated administration. Future studies should assess the transport efficiency of different ligands and peptide strategies in orthotopic GBM models and explore long-term biodistribution and neurotoxicity to inform clinical readiness.

### 3.8. Curcumin Nanocarrier to Modulate Autophagy in GBM Treatment

**Figure 4:**
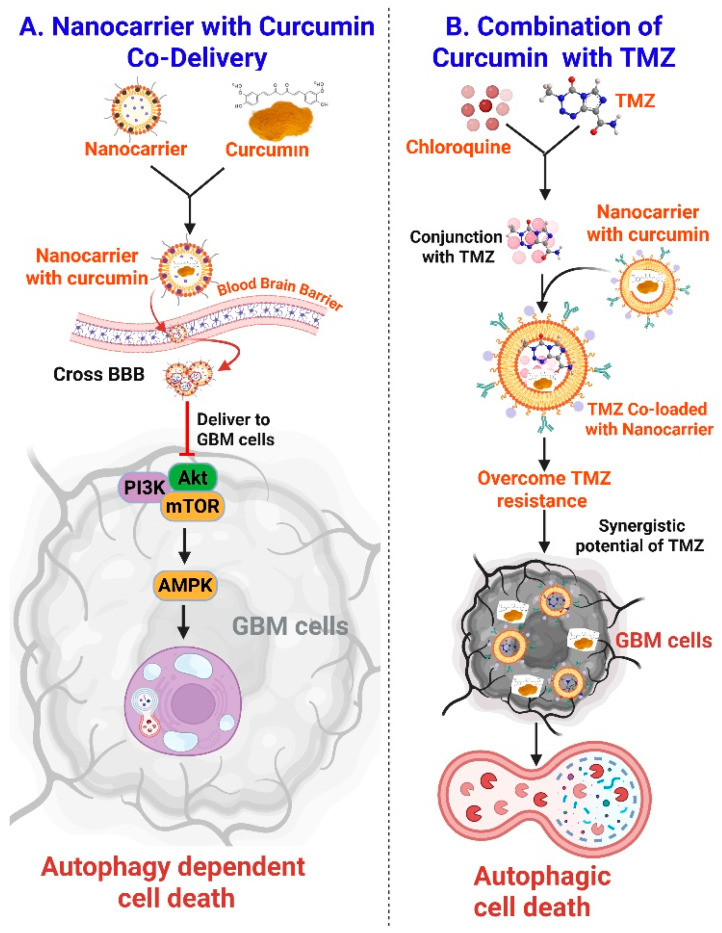
**Curcumin nanocarrier-mediated modulation of autophagy in glioblastoma therapy.** (**A**) Nanocarrier-based curcumin delivery enhances blood–brain barrier penetration and targets glioblastoma cells. Upon cellular uptake, curcumin modulates the PI3K/AKT/mTOR and AMPK signaling pathways, triggering autophagy-dependent cell death. (**B**) Co-delivery of curcumin with TMZ using nanocarrier systems or in combination with autophagy modulators like chloroquine helps overcome TMZ resistance. This combinatorial approach synergistically induces autophagic cell death, suppressing GBM progression and enhancing therapeutic efficacy. The figure was created and modified using the BioRender.com online commercial platform.

Liposomes, polymeric nanoparticles, dendrimers, solid lipid nanoparticles, and nanomicelles are examples of nanocarriers that provide enhanced curcumin encapsulation, defense against metabolic breakdown, and extended systemic circulation. Through passive targeting (increased permeability and retention effect) or active targeting (using ligands against overexpressed GBM markers), these carriers help curcumin permeate the blood–brain barrier and enable targeted delivery to tumor tissues [4]. When administered intracellularly, nanocurcumin effectively inhibits the PI3K/AKT/mTOR pathway and activates AMPK to control autophagy (Figure 4A) [103]. As a result, autophagic signaling cascades are initiated, which increases the production of autophagosomes, LC3B lipidation, and p62 degradation, all of which are indicators of autophagic flux [104]. Increased autophagy encourages ADCD, especially in cells that resemble gliomas and are frequently resistant to traditional treatment. Moreover, chemotherapeutic drugs like temozolomide or autophagy inhibitors (Chloroquine) co-loaded into multifunctional nanocarriers with curcumin can work in concert to improve therapeutic efficacy and overcome resistance (Figure 4B) [105]. In the acidic tumor microenvironment, smart nanocarrier technologies also provide regulated, stimuli-responsive medication release [106]. Thus, by enhancing autophagy-mediated tumor cell death and enhancing therapeutic results in drug-resistant glioblastoma, curcumin-loaded nanocarriers offer a viable strategy for GBM treatment.

## 4. Recent Preclinical Studies with Curcumin and Nanocurcumin Therapeutic Synergy in Glioblastoma

Both curcumin and its nanoformulations have been shown in preclinical studies to have strong anticancer effects against glioblastoma [107]. These studies demonstrate curcumin’s ability to improve the effectiveness of common treatments including temozolomide (TMZ), radiation, and immunotherapy in addition to its direct cytotoxic effects on glioma cells [108]. One possible approach to overcome therapeutic resistance and enhancing treatment results is the incorporation of nanocurcumin into combinatorial regimens. Preclinical Evaluation of Curcumin and Nanocurcumin in Glioblastoma is presented in Table 1.

### 4.1. GBM Models with Curcumin Monotherapy

Several in vitro investigations have demonstrated that curcumin by itself causes apoptosis, cell cycle arrest, and suppression of cell proliferation in glioblastoma cell lines, including U87, U251, and T98G [113]. Curcumin therapy suppresses oncogenic signaling pathways including NF-κB and PI3K/AKT/mTOR, increases the expression of pro-apoptotic markers like Bax and cleaved caspases, and decreases the expression of anti-apoptotic proteins like Bcl-2 [114]. Significant decreases in tumor volume and angiogenesis have been shown in vivo studies employing orthotopic and subcutaneous xenograft models; however, bioavailability limitations sometimes restrict the effectiveness of treatment.

### 4.2. Synergy with Temozolomide

When used in conjunction with TMZ, the current gold standard for GBM chemotherapy, curcumin has demonstrated encouraging synergistic effects. By inhibiting MGMT (O6-methylguanine-DNA methyltransferase), a DNA repair enzyme that promotes resistance to alkylating drugs, curcumin increases the cytotoxicity caused by TMZ [115]. In GBM xenograft models, co-treatment with curcumin and TMZ has been demonstrated to enhance apoptosis, decrease tumor cell viability, and postpone tumor development [116]. Additionally, curcumin contributes to TMZ sensitivity by inhibiting survival pathways like AKT and STAT3 [28]. Nanocurcumin formulations enhance this interaction by increasing curcumin’s bioavailability and delivery [117]. For example, when TMZ and curcumin-loaded PLGA nanoparticles are administered together, mice show better tumor growth inhibition and survival benefits than when each drug is used alone [118]. These results highlight how nanocurcumin may improve conventional chemotherapy treatments.

### 4.3. Improving Radiotherapy

Due to DNA repair processes, hypoxic microenvironments, and glioma stem-like cells, radiation resistance is a significant challenge in the treatment of glioblastoma. By boosting apoptosis, inhibiting DNA repair pathways, and raising intracellular ROS, curcumin has been shown to radiosensitize GBM cells [119]. Additionally, it prevents radiation from activating survival pathways including STAT3 and NF-κB [120]. Formulations of nanocurcumin enhance these effects by enabling long-term administration to the tumor site [121]. Nanocurcumin pretreatment dramatically increased tumor response to fractionated radiation, decreased recurrence rates, and extended longevity in mouse glioma models [122]. According to these results, curcumin may reduce the effective radiation dose needed for tumor control and act as a natural radiosensitizer.

### 4.4. Combinations of Potential Immunotherapy

Recent research suggests that curcumins have immunomodulatory qualities that could enhance immunotherapy strategies for glioblastoma. In the tumor microenvironment, it has been demonstrated to inhibit regulatory T cells (Tregs), reduce immunosuppressive cytokines (e.g., IL-10, TGF-β), and downregulate PD-L1 expression [123]. These steps may improve the effectiveness of immune checkpoint inhibition and restore T-cell function. Combining nanocurcumin with anti-PD-1/PD-L1 antibodies in GBM models has demonstrated increased infiltration of CD8^+^ cytotoxic T cells and decreased tumor burden [124], albeit this research is still in its early stages. These encouraging findings suggest that nanocurcumin may be used as an adjuvant in immunotherapy treatments in the future.

### 4.5. Combinations with Phytochemicals and Natural Compounds

To increase its therapeutic potential, curcumin has also been tested in conjunction with other natural compounds such piperine, resveratrol, quercetin, and epigallocatechin gallate (EGCG) [125]. There have been reports of synergistic interactions, especially in the control of autophagy, oxidative stress, and mitochondrial dysfunction [126]. Specifically, piperine increases curcumin’s anticancer efficacy and absorption [127]. When incorporated into nanoparticle delivery methods, these combinations become much more potent.

### 4.6. Targeting Glioma Stem Cells

Glioma stem-like cells’ (GSCs’) tenacity is a major factor in tumor recurrence and therapy failure. It has been shown that curcumin can inhibit GSC self-renewal, promote differentiation, and make these cells more sensitive to radiation and TMZ [30]. By increasing GSC absorption and cytotoxicity, nanocurcumin offers a distinct edge when it comes to treating populations that are resistant to treatment [121]. In preclinical experiments, dual-delivery nanoparticle systems that co-encapsulate curcumin, and chemotherapeutic drugs have demonstrated strong anti-GSC effects [128].

## 5. Challenges, Limitations, and Future Directions

Despite the strong preclinical evidence that curcumin and nanocurcumin are effective treatments for glioblastoma, there are still several major obstacles in the way of their successful clinical translation. To promote patient safety, regulatory approval, and therapeutic efficacy in clinical settings, these restrictions need to be seriously addressed.

### 5.1. Limitations in Pharmacokinetics and Bioavailability

Historically, curcumin’s clinical development has been hindered by its low oral bioavailability, fast systemic metabolism, and poor water solubility [129]. Even though nanocarrier systems have significantly improved curcumin’s pharmacokinetic profile, several challenges remain. Differences in drug loading efficiency, release kinetics, and biodistribution across various platforms continue to raise concern [130]. Furthermore, reproducibility and scalability may be hampered by the lack of consistency in formulation techniques and the inadequate long-term stability of certain nanoformulations.

### 5.2. Penetration of the Blood–Brain Barrier (BBB)

In pre-clinical models, nanocarriers have demonstrated enhanced BBB permeability; nonetheless, overcoming the human BBB is still a significant challenge [131]. Translational results are complicated by the physiological differences between human and animal models [132]. Furthermore, it may be difficult for nanoparticles to be functionalized with targeting ligands to accumulate sufficiently and selectively in glioma tissues without causing off-target effects.

### 5.3. Difficulties with Manufacturing and Regulation

Producing clinical grade nanocurcumin necessitates rigorous compliance with Good Manufacturing Practice (GMP) standards. This entails meticulous management of essential concerns, including endotoxin contamination, nanoparticle aggregation, and batch-to-batch variability. The regulatory environment is unclear due to the absence of defined rules for phytochemical-loaded nanocarriers, complicating the licensing procedure. Natural product nanoformulations frequently lack strong pharmacological, toxic, and pharmacokinetic evidence that regulatory agencies like the FDA require [133].

### 5.4. Considerations for Safety and Toxicity

Although curcumin is generally recognized as safe (GRAS) by the FDA and has exhibited minimal toxicity in animal studies, the long-term safety profile of nanocurcumin formulations remains insufficiently understood and warrants further investigation. In organs like the liver, spleen, or kidneys, nanoparticles can build up and cause oxidative damage or unanticipated immunological reactions [134]. Prior to human use, thorough toxicological investigations and in-depth assessments of organ distribution, biodegradability, and clearance are required.

### 5.5. Insufficient Clinical Experiments

A substantial disparity persists between the encouraging outcomes of nanocurcumin in preclinical glioblastoma models and its corroboration in clinical environments. Although numerous early-phase trials have examined curcumin in various cancers, its effectiveness against primary brain tumors has been minimally assessed in clinical investigations [135]. The clinical advantage of nanocurcumin in GBM patients has not yet been shown in any randomized controlled trials (RCTs) [135]. This disparity emphasizes the necessity of carefully planned multicenter studies with clear objectives, suitable dosage schedules, and patient classification based on biomarkers.

### 5.6. Microenvironmental Resistance and Tumor Heterogeneity

Glioblastoma is a very heterogeneous neoplasm distinguished by several molecular subtypes and intricate microenvironmental interactions. In reaction to therapeutic pressure, tumor cells swiftly adjust by reconfiguring signaling networks and enlisting immune and stromal elements. In this situation, curcumin’s pleiotropic activity is beneficial, but resistance mechanisms may still manifest. Strategies for combination therapy need to be logically developed using molecular profiling and real-time evaluation of treatment outcomes [136].

### 5.7. Prospects for the Future Direction

Future studies should focus on designing multifunctional nanoparticles capable of co-delivering curcumin alongside immunotherapeutics, gene-editing tools (such as siRNA or CRISPR), or chemotherapeutic agents. This combinatorial approach may significantly enhance the therapeutic efficacy and precision of glioblastoma treatment [137]. However, creating targeted delivery systems that use external stimuli (such as pH, temperature, or magnetic fields) to regulate drug release, tumor-specific ligands, and receptor-mediated transcytosis. Carrying out thorough research in immunocompetent animals and patient-derived xenografts (PDX) to more closely resemble immunological response and clinical behavior [138]. To assess the safety, ideal dosage, pharmacokinetics, and initial effectiveness of nanocurcumin formulations, early-phase clinical trials are being started, particularly in patients with glioblastoma [139]. Moreover, using omics and bioinformatics technology to find predictive biomarkers for resistance and response and to tailor therapeutic strategies [140].

## 6. Critical Perspectives on Curcumin-Loaded Nanocarriers for GBM Therapy

To improve the scientific merit of the Nanocarriers for the delivery of curcumin for glioblastoma, a more balanced approach is needed in the different sections. Focusing on formulation details does not sufficiently compare their therapeutic relevance or mechanistic benefits. Different studies vary substantially regarding which delivery platform is superior. For instance, while polymeric nanoparticles have prolonged release and are well-studied, there are concerns about reproducibility and immunogenicity. On the other hand, liposomes have better biocompatibility but often suffer from limited drug loading and rapid clearance. Dendrimers, despite their extensive functionalization, are associated with toxicity in higher generations. These nuances are not sufficiently explored. In addition, there are limited comparative efficacy data, with few head-to-head preclinical studies demonstrating the differences in tumor penetration, blood–brain barrier crossing, and survival benefits. The identification of such gaps could guide the focus of future research more incisively. Furthermore, while many formulations show promise in vitro or in preclinical models, very few have progressed to early-stage clinical trials. A greater emphasis on translational readiness, including aspects such as scalability, regulatory challenges, and pharmacokinetics, would better inform the readers of which nanocarrier approaches hold the most therapeutic promise for glioblastoma.

## 7. Conclusions

A promising treatment approach for glioblastoma is curcumin and its nanoformulations, which overcome the shortcomings of existing therapies while providing multi-targeted anticancer effects. Curcumin promotes apoptosis, inhibits angiogenesis, and makes tumor cells more sensitive to temozolomide and radiation while also modulating important molecular pathways implicated in the pathophysiology of GBM, such as PI3K/AKT/mTOR, NF-κB, and STAT3 [141]. Its therapeutic use has been hindered, nevertheless, by its poor solubility, low bioavailability, and limited blood–brain barrier penetration. Liposomes, polymeric nanoparticles, and dendrimers are examples of nanocarrier-based delivery systems that improve curcumin’s stability, BBB permeability, and tumor-specific targeting [142]. Preclinical evidence for the activity of nanocurcumin as a single agent and its combination with TMZ and immune checkpoint inhibitors is compelling. Despite recent advances, there is an urgent need for increased translational work, standardization of formulation protocols and thoughtful clinical trial design. If well-designed, nanocurcumin has the potential to be a meaningful adjunct to current treatment regimens for glioblastoma, with the goal of improving outcomes and quality of life for these patients.

## Figures and Tables

**Table 1:** Therapeutic and preclinical evaluation of nanocurcumin in GBM.

Therapeutic Strategy	Preclinical Findings	Role of Nanocurcumin	Refs.
**Curcumin Monotherapy in GBM Models**	Induces apoptosis, cell cycle arrest, and inhibits proliferation in U87, U251, T98G cell lines; suppresses NF-κB and PI3K/AKT/mTOR pathways; increases Bax, cleaved caspases; reduces Bcl-2. In vivo, it reduces tumor size and angiogenesis.	Enhances curcumin stability and BBB penetration; improves overall bioavailability.	[30]
**Combination with Temozolomide (TMZ)**	Synergistically enhances TMZ effects by inhibiting MGMT expression and suppressing AKT/STAT3 pathways; increases apoptosis and reduces tumor growth in xenograft models.	Increases delivery efficiency; co-loaded PLGA nanoparticles of curcumin and TMZ show superior tumor inhibition and survival benefits compared to monotherapy.	[109]
**Enhancing Radiotherapy**	Radiosensitizes GBM cells by increasing ROS levels, suppressing DNA repair, and inhibiting NF-κB/STAT3 activation; enhances radiation-induced apoptosis.	Enables sustained delivery and localized tumor accumulation; increases radiotherapy response, decreases recurrence, and improves survival in mouse models.	[110]
**Combination with Immunotherapy**	Suppresses Tregs, IL-10, TGF-β; downregulates PD-L1; promotes CD8+ T-cell infiltration; enhances immune checkpoint blockade efficacy.	Facilitates co-delivery with anti-PD-1/PD-L1 antibodies; boosts immune activation and reduces tumor burden in GBM preclinical models.	[111]
**Combination with Natural Compounds**	Synergizes with agents like piperine, resveratrol, quercetin, EGCG; regulates autophagy, oxidative stress, and mitochondrial dysfunction; piperine improves curcumin uptake.	Co-encapsulation improves curcumin absorption and therapeutic efficacy through enhanced stability and targeted delivery.	[112]
**Targeting Glioma Stem Cells (GSCs)**	Inhibits self-renewal, promotes differentiation, and sensitizes GSCs to TMZ and radiation; shows cytotoxicity in resistant cell populations.	Dual drug nanocarriers with curcumin improve uptake and cytotoxicity in GSCs demonstrate enhanced anti-tumor effects in resistant GBM subpopulations.	[6]

## Data Availability

No new data were created or analyzed in this study.

## References

[1] Sipos D., Raposa B.L., Freihat O., Simon M., Mekis N., Cornacchione P., Kovács Á. (2025). Glioblastoma: Clinical presentation, multidisciplinary management, and Long-Term outcomes. Cancers.

[2] Bijalwan G., Shrivastav A.K., Mallik S., Dubey M.K. (2024). Glioblastoma multiforme-a rare type of cancer: A narrative review. Cancer Res. Stat. Treat..

[3] Munagama C.L., Rajendiran V., Silva S. (2024). A case report on a common tumour with an uncommon presentation: Glioblastoma. Cureus.

[4] Duan M., Cao R., Yang Y., Chen X., Liu L., Ren B., Wang L., Goh B.-C. (2024). Blood–brain barrier conquest in glioblastoma nanomedicine: Strategies, clinical advances, and emerging challenges. Cancers.

[5] Roney M., Huq A.M., Rullah K., Zamri N.B., Mohd Aluwi M.F.F. (2025). Curcumin, a bioactive compound of Turmeric (*Curcuma longa*) and its derivatives as α-amylase and α-glucosidase inhibitors. Cell Biochem. Biophys..

[6] Nowacka A., Ziółkowska E., Smuczyński W., Bożiłow D., Śniegocki M. (2025). Potential of Curcumin and Its Analogs in Glioblastoma Therapy. Antioxidants.

[7] Fatima F., Chourasiya N.K., Mishra M., Kori S., Pathak S., Das R., Kashaw V., Iyer A.K., Kashaw S.K. (2024). Curcumin and its derivatives targeting multiple signaling pathways to elicit anticancer activity: A comprehensive perspective. Curr. Med. Chem..

[8] Wong S.C., Kamarudin M.N.A., Naidu R. (2021). Anticancer mechanism of curcumin on human glioblastoma. Nutrients.

[9] Zoi V., Galani V., Vartholomatos E., Zacharopoulou N., Tsoumeleka E., Gkizas G., Bozios G., Tsekeris P., Chousidis I., Leonardos I. (2021). Curcumin and radiotherapy exert synergistic anti-glioma effect in vitro. Biomedicines.

[10] Bagherian A., Mardani R., Roudi B., Taghizadeh M., Banfshe H.R., Ghaderi A., Davoodvandi A., Shamollaghamsari S., Hamblin M.R., Mirzaei H. (2020). Combination therapy with nanomicellar-curcumin and temozolomide for in vitro therapy of glioblastoma multiforme via Wnt signaling pathways. J. Mol. Neurosci..

[11] Wen C., Cao L., Yu Z., Liu G., Zhang J., Xu X. (2024). Advances in lipo-solubility delivery vehicles for curcumin: Bioavailability, precise targeting, possibilities and challenges. Crit. Rev. Food Sci. Nutr..

[12] Habeeb M., Vengateswaran H.T., You H.W., Saddhono K., Aher K.B., Bhavar G.B. (2024). Nanomedicine facilitated cell signaling blockade: Difficulties and strategies to overcome glioblastoma. J. Mater. Chem. B.

[13] Kirit E., Gokce C., Altun B., Yilmazer A.e. (2025). Nanotherapeutic Strategies for Overcoming the Blood–Brain Barrier: Applications in Disease Modeling and Drug Delivery. ACS Omega.

[14] Khatoon S., Kalam N. (2025). Mechanistic insight of curcumin: A potential pharmacological candidate for epilepsy. Front. Pharmacol..

[15] Musielak E., Krajka-Kuźniak V. (2025). Lipidic and Inorganic Nanoparticles for Targeted Glioblastoma Multiforme Therapy: Advances and Strategies. Micro.

[16] Wu Y., Moonshi S.S., Ta H.T. (2025). Advancements in Using Polymeric Nanoparticles for Blood–Brain Barrier Penetration in Neurological Disorders. ACS Appl. Bio Mater..

[17] Shetty N.P., Prabhakaran M., Srivastava A.K. (2021). Pleiotropic nature of curcumin in targeting multiple apoptotic-mediated factors and related strategies to treat gastric cancer: A review. Phytother. Res..

[18] Gautam M., Gabrani R. (2024). Current combinatorial therapeutic aspects: The future prospect for glioblastoma treatment. Curr. Med. Sci..

[19] Li X., Wu C., Chen N., Gu H., Yen A., Cao L., Wang E., Wang L. (2016). PI3K/Akt/mTOR signaling pathway and targeted therapy for glioblastoma. Oncotarget.

[20] Ouyang L., Shi Z., Zhao S., Wang F.T., Zhou T.T., Liu B., Bao J.K. (2012). Programmed cell death pathways in cancer: A review of apoptosis, autophagy and programmed necrosis. Cell Prolif..

[21] Wang Z., Liu F., Liao W., Yu L., Hu Z., Li M., Xia H. (2020). Curcumin suppresses glioblastoma cell proliferation by p-AKT/mTOR pathway and increases the PTEN expression. Arch. Biochem. Biophys..

[22] Zoi V., Kyritsis A.P., Galani V., Lazari D., Sioka C., Voulgaris S., Alexiou G.A. (2024). The role of curcumin in cancer: A focus on the PI3K/Akt pathway. Cancers.

[23] Rai M., Pandit R., Paralikar P., Nagaonkar D., Rehman F., Alves dos Santos C. (2017). Pharmaceutical applications of curcumin-loaded nanoparticles. Nanotechnology Applied to Pharmaceutical Technology.

[24] Deng X.-Z., Geng S.-S., Luo M., Chai J.-J., Xu Y., Chen C.-L., Qiu L., Ke Q., Duan Q.-W., Song S.-M. (2020). Curcumin potentiates laryngeal squamous carcinoma radiosensitivity via NF-ΚB inhibition by suppressing IKKγ expression. J. Recept. Signal Transduct..

[25] Sarkar S., Patranabis S. (2024). Immunomodulatory signalling networks in glioblastoma multiforme: A comprehensive review of therapeutic approaches. Hum. Cell.

[26] Oeckinghaus A., Ghosh S. (2009). The NF-κB family of transcription factors and its regulation. Cold Spring Harb. Perspect. Biol..

[27] Piperi C., Papavassiliou K.A., Papavassiliou A.G. (2019). Pivotal role of STAT3 in shaping glioblastoma immune microenvironment. Cells.

[28] Golmohammadi M., Zamanian M.Y., Al-Ani A.M., Jabbar T.L., Kareem A.K., Aghaei Z.H., Tahernia H., Hjazi A., Jissir S.A.R., Hakimizadeh E. (2024). Targeting STAT3 signaling pathway by curcumin and its analogues for breast cancer: A narrative review. Anim. Models Exp. Med..

[29] Liu M., Wang J., Song Z., Pei Y. (2025). Regulation mechanism of curcumin mediated inflammatory pathway and its clinical application: A review. Front. Pharmacol..

[30] Ryskalin L., Biagioni F., Busceti C.L., Lazzeri G., Frati A., Fornai F. (2020). The multi-faceted effect of curcumin in glioblastoma from rescuing cell clearance to autophagy-independent effects. Molecules.

[31] Kadiyala P., Gregory J.V., Lowenstein P.R., Lahann J., Castro M.G. (2021). Targeting gliomas with STAT3-silencing nanoparticles. Mol. Cell. Oncol..

[32] Sidhar H., Giri R.K. (2017). Induction of Bex genes by curcumin is associated with apoptosis and activation of p53 in N2a neuroblastoma cells. Sci. Rep..

[33] Bock F.J., Tait S.W. (2020). Mitochondria as multifaceted regulators of cell death. Nat. Rev. Mol. Cell Biol..

[34] Salucci S., Bavelloni A., Stella A.B., Fabbri F., Vannini I., Piazzi M., Volkava K., Scotlandi K., Martinelli G., Faenza I. (2023). The cytotoxic effect of curcumin in rhabdomyosarcoma is associated with the modulation of AMPK, AKT/mTOR, STAT, and p53 signaling. Nutrients.

[35] Ramli I., Cheriet T., Posadino A.M., Giordo R., Fenu G., Nwachukwu K.C., Oyewole O.A., Adetunji C.O., Calina D., Sharifi-Rad J. (2024). Modulating the p53-MDM2 pathway: The therapeutic potential of natural compounds in cancer treatment. EXCLI J..

[36] Zepeda-Quiróz I., Sánchez-Barrera H., Colín-Val Z., Robledo-Cadena D.X., Rodríguez-Enríquez S., López-Marure R. (2020). Curcumin promotes oxidative stress, apoptosis and autophagy in H9c2 rat cardiomyoblasts. Mol. Cell. Toxicol..

[37] Nainggolan S.I., Rajuddin R., Kamarlis R.K., Hambal M., Frengki F. (2025). In silico study of the potential of curcumin and its derivatives for increasing wild-type p53 expression and improving the function of p53 mutant R273H. Vet. World.

[38] Salehi A.M., Hajjari M., Pourreza N., Khodadadi A. (2025). Investigating How Nano-Curcumin Affects the Expression of the p53 Gene and Inhibits the Cell Cycle in the A549 Lung Cancer Cell Line. Jentashapir J. Cell. Mol. Biol..

[39] Cui J., Li H., Zhang T., Lin F., Chen M., Zhang G., Feng Z. (2025). Research progress on the mechanism of curcumin anti-oxidative stress based on signaling pathway. Front. Pharmacol..

[40] Wang F., Dezfouli A.B., Khosravi M., Sievert W., Stangl S., Schwab M., Wu Z., Steiger K., Ma H., Multhoff G. (2023). Cannabidiol-induced crosstalk of apoptosis and macroautophagy in colorectal cancer cells involves p53 and Hsp70. Cell Death Discov..

[41] Alizadeh M., Kheirouri S. (2019). Curcumin reduces malondialdehyde and improves antioxidants in humans with diseased conditions: A comprehensive meta-analysis of randomized controlled trials. BioMedicine.

[42] Ji S., Sun R., Xu K., Man Z., Ji J., Pu Y., Yin L., Zhang J., Pu Y. (2019). Prodigiosin induces apoptosis and inhibits autophagy via the extracellular signal-regulated kinase pathway in K562 cells. Toxicol. Vitr..

[43] Park J.-Y., Choi Y., Kim H.-D., Kuo H.-H., Chang Y.-C., Kim C.-H. (2025). Matrix Metalloproteinases and Their Inhibitors in the Pathogenesis of Epithelial Differentiation, Vascular Disease, Endometriosis, and Ocular Fibrotic Pterygium. Int. J. Mol. Sci..

[44] Wroński P., Wroński S., Kurant M., Malinowski B., Wiciński M. (2021). Curcumin may prevent basement membrane disassembly by matrix metalloproteinases and progression of the bladder cancer. Nutrients.

[45] Senft C., Polacin M., Priester M., Seifert V., Kögel D., Weissenberger J. (2010). The nontoxic natural compound Curcumin exerts anti-proliferative, anti-migratory, and anti-invasive properties against malignant gliomas. BMC Cancer.

[46] Zanotto-Filho A., Braganhol E., Klafke K., Figueiró F., Terra S.R., Paludo F.J., Morrone M., Bristot I.J., Battastini A.M., Forcelini C.M. (2015). Autophagy inhibition improves the efficacy of curcumin/temozolomide combination therapy in glioblastomas. Cancer Lett..

[47] Yaroshenko M., Christoff M., Ścibiorski M., Surowiec K., Jakubowicz-Gil J., Sumorek-Wiadro J. (2025). Natural Compounds That Target Glioma Stem Cells. NeuroSci.

[48] Naujokat C., McKee D.L. (2021). The “Big Five” phytochemicals targeting cancer stem cells: Curcumin, EGCG, sulforaphane, resveratrol and genistein. Curr. Med. Chem..

[49] Mattei V., Santilli F., Martellucci S., Delle Monache S., Fabrizi J., Colapietro A., Angelucci A., Festuccia C. (2021). The importance of tumor stem cells in glioblastoma resistance to therapy. Int. J. Mol. Sci..

[50] Peter K., Gandhi P., Kar S.K. (2025). Curcumin and 2-DG synergistically target the glio-oncogenesis trigger IL-6 and down-regulate the stemness in glioblastoma model in-vitro. Adv. Tradit. Med..

[51] Sabu A., Liu T.-I., Ng S.S., Doong R.-A., Huang Y.-F., Chiu H.-C. (2022). Nanomedicines targeting glioma stem cells. ACS Appl. Mater. Interfaces.

[52] Gersey Z.C., Rodriguez G.A., Barbarite E., Sanchez A., Walters W.M., Ohaeto K.C., Komotar R.J., Graham R.M. (2017). Curcumin decreases malignant characteristics of glioblastoma stem cells via induction of reactive oxygen species. BMC Cancer.

[53] Zhang M., Xiang C., Niu R., He X., Luo W., Liu W., Gu R. (2025). Liposomes as versatile agents for the management of traumatic and nontraumatic central nervous system disorders: Drug stability, targeting efficiency, and safety. Neural Regen. Res..

[54] Rahman M.A., Jalouli M., Al-Zharani M., Hoque Apu E., Harrath A.H. (2025). Mechanistic Insights into Autophagy-Dependent Cell Death (ADCD): A Novel Avenue for Cancer Therapy. Cells.

[55] Shakeri A., Cicero A.F., Panahi Y., Mohajeri M., Sahebkar A. (2019). Curcumin: A naturally occurring autophagy modulator. J. Cell. Physiol..

[56] Wang N., Feng T., Liu X., Liu Q. (2020). Curcumin inhibits migration and invasion of non-small cell lung cancer cells through up-regulation of miR-206 and suppression of PI3K/AKT/mTOR signaling pathway. Acta Pharm..

[57] Han J., Pan X.-Y., Xu Y., Xiao Y., An Y., Tie L., Pan Y., Li X.-J. (2012). Curcumin induces autophagy to protect vascular endothelial cell survival from oxidative stress damage. Autophagy.

[58] Fabianowska-Majewska K., Kaufman-Szymczyk A., Szymanska-Kolba A., Jakubik J., Majewski G., Lubecka K. (2021). Curcumin from turmeric rhizome: A potential modulator of DNA methylation machinery in breast cancer inhibition. Nutrients.

[59] Zhang L., Yang Y., Li Y., Wang C., Bian C., Wang H., Wang F. (2025). Epigenetic regulation of histone modifications in glioblastoma: Recent advances and therapeutic insights. Biomark. Res..

[60] Fu S., Kurzrock R. (2010). Development of curcumin as an epigenetic agent. Cancer.

[61] Miles F.L., Mashchak A., Filippov V., Orlich M.J., Duerksen-Hughes P., Chen X., Wang C., Siegmund K., Fraser G.E. (2020). DNA methylation profiles of vegans and non-vegetarians in the adventist health study-2 cohort. Nutrients.

[62] Sminia P., van den Berg J., van Kootwijk A., Hageman E., Slotman B.J., Verbakel W.F. (2021). Experimental and clinical studies on radiation and curcumin in human glioma. J. Cancer Res. Clin. Oncol..

[63] Amini A., Khadivar P., Ahmadnia A., Alipour M., Majeed M., Jamialahmadi T., Sathyapalan T., Sahebkar A. (2021). Role of curcumin in regulating long noncoding RNA expression in cancer. Pharmacological Properties of Plant-Derived Natural Products and Implications for Human Health.

[64] Fu X., He Y., Li M., Huang Z., Najafi M. (2021). Targeting of the tumor microenvironment by curcumin. Biofactors.

[65] Admasu T.D., Yu J.S. (2025). Harnessing Immune Rejuvenation: Advances in Overcoming T Cell Senescence and Exhaustion in Cancer Immunotherapy. Aging Cell.

[66] Hayakawa T., Yaguchi T., Kawakami Y. (2020). Enhanced anti-tumor effects of the PD-1 blockade combined with a highly absorptive form of curcumin targeting STAT3. Cancer Sci..

[67] Jiang M., Qi Y., Huang W., Lin Y., Li B. (2022). Curcumin Reprograms TAMs from a protumor phenotype towards an antitumor phenotype via inhibiting MAO-A/STAT6 Pathway. Cells.

[68] Parker J.M., Zhao L., Mayberry T.G., Cowan B.C., Wakefield M.R., Fang Y. (2025). From Spice to Survival: The Emerging Role of Curcumin in Cancer Immunotherapy. Cancers.

[69] Saberian E., Jenčová J., Jenča A., Jenča A., Petrášová A., Jenča J., Akbarzadehkhayavi A. (2025). Combination Therapy of Curcumin and Cisplatin Encapsulated in Niosome Nanoparticles for Enhanced Oral Cancer Treatment. Indian. J. Clin. Biochem..

[70] Zheng B., McClements D.J. (2020). Formulation of More Efficacious Curcumin Delivery Systems Using Colloid Science: Enhanced Solubility, Stability, and Bioavailability. Molecules.

[71] Obeid M.A., Alsaadi M., Aljabali A.A. (2023). Recent updates in curcumin delivery. J. Liposome Res..

[72] Tashima T. (2020). Smart Strategies for Therapeutic Agent Delivery into Brain across the Blood-Brain Barrier Using Receptor-Mediated Transcytosis. Chem. Pharm. Bull..

[73] Rafiee Z., Nejatian M., Daeihamed M., Jafari S.M. (2019). Application of curcumin-loaded nanocarriers for food, drug and cosmetic purposes. Trends Food Sci. Technol..

[74] Santhanakrishnan K.R., Koilpillai J., Narayanasamy D., Santhanakrishnan K. (2024). PEGylation in pharmaceutical development: Current status and emerging trends in macromolecular and immunotherapeutic drugs. Cureus.

[75] Larrañeta E., Stewart S., Ervine M., Al-Kasasbeh R., Donnelly R.F. (2018). Hydrogels for Hydrophobic Drug Delivery. Classification, Synthesis and Applications. J. Funct. Biomater..

[76] Chopra H., Dey P.S., Das D., Bhattacharya T., Shah M., Mubin S., Maishu S.P., Akter R., Rahman M.H., Karthika C. (2021). Curcumin Nanoparticles as Promising Therapeutic Agents for Drug Targets. Molecules.

[77] Karatug Kacar A., Sak R., Nurdogan A.N., Ergin Kızılcay G., Bahadori F. (2025). Dual-targeted protein-coated PLGA nanoparticles for pancreatic cancer therapy: A novel approach using esculetin and curcumin. Naunyn Schmiedebergs Arch. Pharmacol..

[78] Hafez Ghoran S., Calcaterra A., Abbasi M., Taktaz F., Nieselt K., Babaei E. (2022). Curcumin-based nanoformulations: A promising adjuvant towards cancer treatment. Molecules.

[79] Tan X., Kim G., Lee D., Oh J., Kim M., Piao C., Lee J., Lee M.S., Jeong J.H., Lee M. (2018). A curcumin-loaded polymeric micelle as a carrier of a microRNA-21 antisense-oligonucleotide for enhanced anti-tumor effects in a glioblastoma animal model. Biomater. Sci..

[80] Aggarwal K., Joshi S., Jindal P., Patel P., Das Kurmi B. (2025). Solid Lipid Nanoparticles: An Innovative Drug Delivery System for Enhanced Bioavailability and Targeted Therapy. AAPS PharmSciTech.

[81] Zhang H., van Os W.L., Tian X., Zu G., Ribovski L., Bron R., Bussmann J., Kros A., Liu Y., Zuhorn I.S. (2021). Development of curcumin-loaded zein nanoparticles for transport across the blood-brain barrier and inhibition of glioblastoma cell growth. Biomater. Sci..

[82] Iturrioz-Rodríguez N., Sampron N., Matheu A. (2023). Current advances in temozolomide encapsulation for the enhancement of glioblastoma treatment. Theranostics.

[83] Fu Z., Chen X., Guan S., Yan Y., Lin H., Hua Z.-C. (2015). Curcumin inhibits angiogenesis and improves defective hematopoiesis induced by tumor-derived VEGF in tumor model through modulating VEGF-VEGFR2 signaling pathway. Oncotarget.

[84] Ciftci F., Özarslan A.C., Kantarci I.C., Yelkenci A., Tavukcuoglu O., Ghorbanpour M. (2025). Advances in drug targeting, drug delivery, and nanotechnology applications: Therapeutic significance in cancer treatment. Pharmaceutics.

[85] McLoughlin C.D., Nevins S., Stein J.B., Khakbiz M., Lee K.B. (2024). Overcoming the Blood-Brain Barrier: Multifunctional Nanomaterial-Based Strategies for Targeted Drug Delivery in Neurological Disorders. Small Sci..

[86] Li D., Fan Y., Shen M., Bányai I., Shi X. (2019). Design of dual drug-loaded dendrimer/carbon dot nanohybrids for fluorescence imaging and enhanced chemotherapy of cancer cells. J. Mater. Chem. B.

[87] Maiti P., Scott J., Sengupta D., Al-Gharaibeh A., Dunbar G.L. (2019). Curcumin and Solid Lipid Curcumin Particles Induce Autophagy, but Inhibit Mitophagy and the PI3K-Akt/mTOR Pathway in Cultured Glioblastoma Cells. Int. J. Mol. Sci..

[88] Dhungel L., Rowsey M.E., Harris C., Raucher D. (2024). Synergistic Effects of Temozolomide and Doxorubicin in the Treatment of Glioblastoma Multiforme: Enhancing Efficacy through Combination Therapy. Molecules.

[89] Janaszewska A., Lazniewska J., Trzepiński P., Marcinkowska M., Klajnert-Maculewicz B. (2019). Cytotoxicity of Dendrimers. Biomolecules.

[90] Cerqueira R., Domingues C., Veiga F., Jarak I., Figueiras A. (2024). Development and Characterization of Curcumin-Loaded TPGS/F127/P123 Polymeric Micelles as a Potential Therapy for Colorectal Cancer. Int. J. Mol. Sci..

[91] Raucher D., Dragojevic S., Ryu J. (2018). Macromolecular Drug Carriers for Targeted Glioblastoma Therapy: Preclinical Studies, Challenges, and Future Perspectives. Front. Oncol..

[92] Liu Y., Zhang Y., Li H., Hu T.Y. (2025). Recent advances in the bench-to-bedside translation of cancer nanomedicines. Acta Pharm. Sin. B.

[93] Dragoj M., Stojkovska J., Stanković T., Dinić J., Podolski-Renić A., Obradović B., Pešić M. (2021). Development and Validation of a Long-Term 3D Glioblastoma Cell Culture in Alginate Microfibers as a Novel Bio-Mimicking Model System for Preclinical Drug Testing. Brain Sci..

[94] Israel L.L., Galstyan A., Holler E., Ljubimova J.Y. (2020). Magnetic iron oxide nanoparticles for imaging, targeting and treatment of primary and metastatic tumors of the brain. J. Control. Release.

[95] Aborig M., Alsefaou M., Osei E., Wettig S. (2025). Engineered dual-functional gold nanoparticles enhance radiosensitization in prostate cancer cells: Synergistic action of curcumin and gold. Nanoscale Adv..

[96] Ghazi R., Ibrahim T.K., Nasir J.A., Gai S., Ali G., Boukhris I., Rehman Z. (2025). Iron oxide based magnetic nanoparticles for hyperthermia, MRI and drug delivery applications: A review. RSC Adv..

[97] Alharbi S.K., Alsehli B.R., AlSuhaimi A.O., Thumayri K.A., AlMohaimadi K.M., Mehdar Y.T.H., Almalki M.A., Hussein B.H.M. (2025). Mesoporous Silica Nanoparticles Functionalized with Bisphenol A for Dispersive Solid-Phase Extraction of 3-Chloroaniline from Water Matrices: Material Synthesis and Sorption Optimization. Nanomater..

[98] Song J., Lu C., Leszek J., Zhang J. (2021). Design and Development of Nanomaterial-Based Drug Carriers to Overcome the Blood-Brain Barrier by Using Different Transport Mechanisms. Int. J. Mol. Sci..

[99] Li C., Zhou L., Yin X. (2024). Pathophysiological aspects of transferrin-A potential nano-based drug delivery signaling molecule in therapeutic target for varied diseases. Front. Pharmacol..

[100] Ghorai S.M., Deep A., Magoo D., Gupta C., Gupta N. (2023). Cell-Penetrating and Targeted Peptides Delivery Systems as Potential Pharmaceutical Carriers for Enhanced Delivery across the Blood-Brain Barrier (BBB). Pharmaceutics.

[101] Paul A., Collins M.G., Lee H.Y. (2022). Gene Therapy: The Next-Generation Therapeutics and Their Delivery Approaches for Neurological Disorders. Front. Genome Ed..

[102] Wahengbam G.S., Nirmal S., Nandwana J., Kar S., Kumari V., Mishra R., Singh A. (2025). Polymeric Nanoparticles Revolutionizing Brain Cancer Therapy: A Comprehensive Review of Strategies and Advances. Crit. Rev. Ther. Drug Carr. Syst..

[103] Khayatan D., Razavi S.M., Arab Z.N., Nasoori H., Fouladi A., Pasha A.V.K., Butler A.E., Karav S., Momtaz S., Abdolghaffari A.H. (2025). Targeting mTOR with curcumin: Therapeutic implications for complex diseases. Inflammopharmacology.

[104] Tian S., Wu L., Zheng H., Zhong X., Yu X., Wu W. (2023). Identification of autophagy-related genes in neuropathic pain through bioinformatic analysis. Hereditas.

[105] Wang C., Li Q., Xiao J., Liu Y. (2023). Nanomedicine-based combination therapies for overcoming temozolomide resistance in glioblastomas. Cancer Biol. Med..

[106] Zhou W., Jia Y., Liu Y., Chen Y., Zhao P. (2022). Tumor Microenvironment-Based Stimuli-Responsive Nanoparticles for Controlled Release of Drugs in Cancer Therapy. Pharmaceutics.

[107] Wahnou H., El Kebbaj R., Liagre B., Sol V., Limami Y., Duval R.E. (2025). Curcumin-Based Nanoparticles: Advancements and Challenges in Tumor Therapy. Pharmaceutics.

[108] Afshari A.R., Sanati M., Aminyavari S., Keshavarzi Z., Ahmadi S.S., Oroojalian F., Karav S., Sahebkar A. (2025). A novel approach to glioblastoma multiforme treatment using modulation of key pathways by naturally occurring small molecules. Inflammopharmacology.

[109] Yin H., Zhou Y., Wen C., Zhou C., Zhang W., Hu X., Wang L., You C., Shao J. (2014). Curcumin sensitizes glioblastoma to temozolomide by simultaneously generating ROS and disrupting AKT/mTOR signaling. Oncol. Rep..

[110] Kim J.Y., Jung C.W., Lee W.S., Kim H.J., Jeong H.J., Park M.J., Jang W.I., Kim E.H. (2022). Interaction of curcumin with glioblastoma cells via high and low linear energy transfer radiation therapy inducing radiosensitization effects. J. Radiat. Res..

[111] Zhong Z., Vong C.T., Chen F., Tan H., Zhang C., Wang N., Cui L., Wang Y., Feng Y. (2022). Immunomodulatory potential of natural products from herbal medicines as immune checkpoints inhibitors: Helping to fight against cancer via multiple targets. Med. Res. Rev..

[112] El-Saadony M.T., Yang T., Korma S.A., Sitohy M., Abd El-Mageed T.A., Selim S., Al Jaouni S.K., Salem H.M., Mahmmod Y., Soliman S.M. (2022). Impacts of turmeric and its principal bioactive curcumin on human health: Pharmaceutical, medicinal, and food applications: A comprehensive review. Front. Nutr..

[113] Arora A., Jain N., Pandey M., Kaul S., Verma R., Gorain B. (2025). Smart Nanometals: An Approach to Transform Brain Cancer Diagnosis and Therapy. Mol. Pharm..

[114] Hedayati N., Safari M.H., Milasi Y.E., Kahkesh S., Farahani N., Khoshnazar S.M., Dorostgou Z., Alaei E., Alimohammadi M., Rahimzadeh P. (2025). Modulation of the PI3K/Akt signaling pathway by resveratrol in cancer: Molecular mechanisms and therapeutic opportunity. Discov. Oncol..

[115] Rao V., Kumar G., Vibhavari R.J.A., Nandakumar K., Thorat N.D., Chamallamudi M.R., Kumar N. (2023). Temozolomide Resistance: A Multifarious Review on Mechanisms Beyond O-6-Methylguanine-DNA Methyltransferase. CNS Neurol. Disord. Drug Targets.

[116] Peter K., Kar S.K., Gothalwal R., Gandhi P. (2021). Curcumin in Combination with Other Adjunct Therapies for Brain Tumor Treatment: Existing Knowledge and Blueprint for Future Research. Int. J. Mol. Cell Med..

[117] Ipar V.S., Dsouza A., Devarajan P.V. (2019). Enhancing Curcumin Oral Bioavailability Through Nanoformulations. Eur. J. Drug Metab. Pharmacokinet..

[118] Keshavarz Shahbaz S., Koushki K., Izadi O., Penson P.E., Sukhorukov V.N., Kesharwani P., Sahebkar A. (2024). Advancements in curcumin-loaded PLGA nanoparticle delivery systems: Progressive strategies in cancer therapy. J. Drug Target..

[119] Zoi V., Galani V., Tsekeris P., Kyritsis A.P., Alexiou G.A. (2022). Radiosensitization and Radioprotection by Curcumin in Glioblastoma and Other Cancers. Biomedicines.

[120] Farhood B., Mortezaee K., Goradel N.H., Khanlarkhani N., Salehi E., Nashtaei M.S., Najafi M., Sahebkar A. (2019). Curcumin as an anti-inflammatory agent: Implications to radiotherapy and chemotherapy. J. Cell Physiol..

[121] Tagde P., Tagde P., Islam F., Tagde S., Shah M., Hussain Z.D., Rahman M.H., Najda A., Alanazi I.S., Germoush M.O. (2021). The Multifaceted Role of Curcumin in Advanced Nanocurcumin Form in the Treatment and Management of Chronic Disorders. Molecules.

[122] Shahcheraghi S.H., Zangui M., Lotfi M., Ghayour-Mobarhan M., Ghorbani A., Jaliani H.Z., Sadeghnia H.R., Sahebkar A. (2019). Therapeutic Potential of Curcumin in the Treatment of Glioblastoma Multiforme. Curr. Pharm. Des..

[123] Mahdi Ghahari S.M., Ajami A., Sadeghizadeh M., Esmaeili Rastaghi A.R., Mahdavi M. (2022). Nanocurcumin as an adjuvant in killed Toxoplasma gondii vaccine formulation: An experience in BALB/c mice. Exp. Parasitol..

[124] Parveen S., Konde D.V., Paikray S.K., Tripathy N.S., Sahoo L., Samal H.B., Dilnawaz F. (2025). Nanoimmunotherapy: The smart trooper for cancer therapy. Explor. Target. Antitumor Ther..

[125] Chimento A., D’Amico M., De Luca A., Conforti F.L., Pezzi V., De Amicis F. (2023). Resveratrol, Epigallocatechin Gallate and Curcumin for Cancer Therapy: Challenges from Their Pro-Apoptotic Properties. Life.

[126] Rainey N.E., Moustapha A., Petit P.X. (2020). Curcumin, a Multifaceted Hormetic Agent, Mediates an Intricate Crosstalk between Mitochondrial Turnover, Autophagy, and Apoptosis. Oxid. Med. Cell Longev..

[127] Bolat Z.B., Islek Z., Demir B.N., Yilmaz E.N., Sahin F., Ucisik M.H. (2020). Curcumin- and Piperine-Loaded Emulsomes as Combinational Treatment Approach Enhance the Anticancer Activity of Curcumin on HCT116 Colorectal Cancer Model. Front. Bioeng. Biotechnol..

[128] Anand P., Kunnumakkara A.B., Newman R.A., Aggarwal B.B. (2007). Bioavailability of curcumin: Problems and promises. Mol. Pharm..

[129] Stohs S.J., Chen O., Ray S.D., Ji J., Bucci L.R., Preuss H.G. (2020). Highly Bioavailable Forms of Curcumin and Promising Avenues for Curcumin-Based Research and Application: A Review. Molecules.

[130] Silvestre F., Santos C., Silva V., Ombredane A., Pinheiro W., Andrade L., Garcia M., Pacheco T., Joanitti G., Luz G. (2023). Pharmacokinetics of Curcumin Delivered by Nanoparticles and the Relationship with Antitumor Efficacy: A Systematic Review. Pharm..

[131] Achar A., Myers R., Ghosh C. (2021). Drug Delivery Challenges in Brain Disorders across the Blood-Brain Barrier: Novel Methods and Future Considerations for Improved Therapy. Biomedicines.

[132] Prabhakar S. (2012). Translational research challenges: Finding the right animal models. J. Investig. Med..

[133] Desai N., Rana D., Patel M., Bajwa N., Prasad R., Vora L.K. (2025). Nanoparticle Therapeutics in Clinical Perspective: Classification, Marketed Products, and Regulatory Landscape. Small.

[134] Ibrahim K.E., Al-Mutary M.G., Bakhiet A.O., Khan H.A. (2018). Histopathology of the Liver, Kidney, and Spleen of Mice Exposed to Gold Nanoparticles. Molecules.

[135] Cacciola N.A., Cuciniello R., Petillo G.D., Piccioni M., Filosa S., Crispi S. (2023). An Overview of the Enhanced Effects of Curcumin and Chemotherapeutic Agents in Combined Cancer Treatments. Int. J. Mol. Sci..

[136] Bristow R.G., Alexander B., Baumann M., Bratman S.V., Brown J.M., Camphausen K., Choyke P., Citrin D., Contessa J.N., Dicker A. (2018). Combining precision radiotherapy with molecular targeting and immunomodulatory agents: A guideline by the American Society for Radiation Oncology. Lancet Oncol..

[137] Hermawan A., Putri H. (2021). Systematic analysis of potential targets of the curcumin analog pentagamavunon-1 (PGV-1) in overcoming resistance of glioblastoma cells to bevacizumab. Saudi Pharm. J..

[138] Okada S., Vaeteewoottacharn K., Kariya R. (2019). Application of Highly Immunocompromised Mice for the Establishment of Patient-Derived Xenograft (PDX) Models. Cells.

[139] Karthikeyan A., Senthil N., Min T. (2020). Nanocurcumin: A Promising Candidate for Therapeutic Applications. Front. Pharmacol..

[140] Olivier M., Asmis R., Hawkins G.A., Howard T.D., Cox L.A. (2019). The Need for Multi-Omics Biomarker Signatures in Precision Medicine. Int. J. Mol. Sci..

[141] Afrashteh Nour M., Rahmati-Yamchi M., Shimia M., Yousefi B., Majidinia M. (2025). Emerging Insights into the PI3K/AKT/mTOR Signaling Pathway and Non-Coding RNA-mediated Drug Resistance in Glioblastoma. Curr. Mol. Med..

[142] Verma R., Rao L., Kumar H., Bansal N., Deep A., Parashar J., Yadav M., Mittal V., Kaushik D. (2025). Applications of Nanomedicine in Brain Tumor Therapy: Nanocarrierbased Drug Delivery Platforms, Challenges, and Perspectives. Recent. Pat. Nanotechnol..

